# Effects of Microwave-Assisted Liquid Hot Water Pretreatment on Chemical Composition and Structure of Moso Bamboo

**DOI:** 10.3389/fbioe.2021.821982

**Published:** 2022-02-07

**Authors:** Jie-Yu Cui, Ning Zhang, Jian-Chun Jiang

**Affiliations:** ^1^ College of Chemical Engineering, Nanjing Forestry University, Nanjing, China; ^2^ Institute of Chemical Industry of Forest Products, Chinese Academy of Forestry, Nanjing, China; ^3^ National Engineering Laboratory for Biomass Chemical Utilization, Nanjing, China; ^4^ Key Laboratory of Chemical Engineering of Forest Products, National Forestry and Grassland Administration, Nanjing, China; ^5^ Key Laboratory of Biomass Energy and Material, Nanjing, China; ^6^ Co-Innovation Center of Efficient Processing and Utilization of Forest Resources, Nanjing Forestry University, Nanjing, China

**Keywords:** Moso bamboo, microwave assisted liquid hot water, pretreatment, structure, hemicellulose

## Abstract

The effects of microwave assisted liquid hot water (MA-LHW) pretreatment on the chemical composition of Moso bamboo were investigated, and the fiber structure of pretreated residues were studied. The results showed that MA-LHW pretreatment had high selectivity for the degradation of hemicellulose in Moso bamboo, and the extracted hemicellulose could be used to prepare xylooligosaccharide through enzyme depolymerization. The degradation rates of cellulose and lignin after MA-LHW pretreatment were only 14.73% and 7.18%, which were significantly lower than those of LHW pretreatment; 155.0 mg/g xylobiose and 61.0 mg/g xylotrisoe can be obtained after enzymatic hydrolysis, and the yield of xylo-oligosaccharide reached 80.59% of the theoretical conversion rate. MA-LHW pretreatment increased the removal of hemicellulose, lignin, and other non-crystalline parts in bamboo materials, and more cellulose with crystalline structure was retained, which increased the CrI value of Moso bamboo by 14.84%. FTIR spectra showed that the characteristic peak intensity of hemicellulose was significantly reduced after MA-LHW pretreatment, which confirmed the selective degradation of hemicellulose by MA-LAW pretreatment. Moreover, MA-LHW pretreatment also destroyed O-H, C-H, C-O-C, and β-glucoside bonds in Moso bamboo fiber, caused by the recombination and synthesis of some groups (-CH_2_ and C=O) of cellulose, hemicellulose, and lignin destroyed under pretreatment conditions.

## 1 Introduction

With the increasing shortage of petrochemical resources and the global energy crisis, the use of renewable biomass resources to produce new materials, fuels, and chemicals has become a research hotspot of many researchers. Moso bamboo is a perennial evergreen plant of Poaceae and Bambusoideae, which is widely distributed in tropical, sub-tropical, and warm temperate regions (Liu et al., 2019). It is one of the most valuable biomasses in the world and has the highest economic value. In addition, it has the advantages of fast growth, strong reproductive capacity, great development, and utilization potential (Wang et al., 2013). In China, Moso bamboo accounts for more than 70% of the country’s forest area, with a total area of 2.92 million hectares and an annual output value of nearly 5.85 billion yuan.

Like other lignocellulosic raw materials, bamboo is composed of cellulose, hemicellulose, and lignin, which interweave with each other to form a complex and dense structure and it is difficult to degrade. The structural system determines that the degradation of any kind of components is bound to be restricted by other components, resulting in the low hydrolysis efficiency of cellulose (Singh et al., 2015). Therefore, it is necessary to pretreat the raw materials, cut off the cellulose structure with cellulose degrading enzyme, release the sugar needed for fermentation, and then use these sugars to further produce bio alcohol liquid fuels such as fuel ethanol, butanol and pentanol, and high value-added chemicals such as xylitol, lactic acid, and so on. One of the difficulties is to find efficient and low-cost pretreatment technology. In contrast, the content of cellulose and lignin in bamboo is higher than that in straw, and it has the higher density and hardness than the straw.

The commonly used pretreatment methods include physical method, chemical method, and biological method (Zhang et al., 2016). Chemical method is widely used because of its advantages of high efficiency and easy industrialization. Like the dilute acid method, it mainly overcomes the obstacle of hemicellulose on the enzymatic hydrolysis in acidic environment such as sulfuric acid, formic acid, and acetic acid by interrupting the molecular chemical bond of polysaccharide. Dilute alkali method mainly uses NaOH, ammonia water, and lime water to dissolve lignin components in the cell wall, so as to increase the contact opportunity between raw cellulose and enzyme and improve the enzymatic hydrolysis rate of cellulose. The liquid hot water (LHW) pretreatment (Zhuang et al., 2016) process uses water as the reaction medium, does not need to add any chemical reagent, and has higher yield of hemicellulose derived sugar (including xylo-oligosaccharides and xylose), higher cellulase hydrolysis rate, and lower yield of degradation products. Compared with supercritical water (temperature higher than 374°C, pressure higher than 22 Mpa), liquid hot water (compressed liquid water between 160°C and 280°C, pressure higher than its saturated vapor pressure) also has good mass transfer performance (low viscosity and high diffusion coefficient). The ion product of liquid hot water is three orders of magnitude larger than that of normal state, which is 10^−11^, that is, the concentration of H^+^ and OH^−^ in neutral water is about 100 times higher than that of normal state (Wang et al., 2016). In this way, the liquid hot water itself will have the function of acid catalysis and alkali catalysis. In addition, its reaction conditions are more mild, are easier to control, and achieve large-scale production.

Microwave-assisted liquid hot water (MA-LHW) method developed on this basis is to use microwave as a heating tool, which can overcome the shortcomings of uneven heating of water, shorten the reaction time, improve the work efficiency, and have the advantages of fast heating speed, uniform heating, no temperature gradient, no lag effect, and so on (Luo et al., 2017). Microwave treatment of materials can produce physical and thermal effects. The physical effect is that microwave radiation produces a continuously changing magnetic field, which leads to the vibration of polar bonds in biomass corresponding to the magnetic field and then provides internal heat to biomass (Chen et al., 2012). This distribution and vibration of polar bonds can accelerate physical, biological, and chemical processes (Ha et al., 2011). Thermal effect is that acetic acid is produced by heat treatment of materials in aqueous solution, which leads to spontaneous hydrolysis of materials in acidic environment. Microwave irradiation can change the ultrastructure of cellulose, remove lignin and hemicellulose, and improve the enzymatic hydrolysis efficiency of cellulose. In this research, the effects of MA-LHW pretreatment on the chemical composition and the structure of Moso bamboo were studied and the extracted hemicellulose was used to produce xylooligosaccharide through further enzyme depolymerization.

## 2 Materials and Methods

### 2.1 Experimental Material

Experimental Moso bamboo powder was supplied by China National Bamboo Research Center. The bamboo powder was sieved through a 0.45 mm mesh screen and dried naturally.

### 2.2 Pretreatment Methods

LHW pretreatment method (Suriyachai et al., 2020): 10 g dried bamboo powder and 100 ml water were mixed into a 250 ml high-pressure reaction kettle. The reaction was run at 200°C for 30 min. After reaction, the mixture was centrifuged, and then the filter cake was washed with distilled water and dried at 105°C until a constant weight.

MA-LHW pretreatment method (Passos et al., 2013): Microwave-assisted liquid hot water pretreatment was carried out with the MWD-520 microwave digestion system which was produced by Shanghai Metash Instruments Co., Ltd.; 1 g dried bamboo powder and 20 ml water were mixed into a 50 ml microwave digestion tube. The reaction was run in the microwave digestion system at 200°C for 30 min. The microwave power was 1,000 W. After reaction, the mixture was centrifuged, and then the filter cake was washed with distilled water and dried at 105°C until a constant weight.

### 2.3 Analysis Method

#### 2.3.1 Composition Analysis

The contents of cellulose, hemicellulose, and lignin in raw materials and pretreated materials were determined according to the method of NREL (Sluiter et al., 2010).

#### 2.3.2 X-Ray Diffraction Analysis

The D8 Foucus X-ray Diffractometer (XRD) produced by Brooke company in Germany was used to determine the relative crystallinity of the samples. The detection wavelength was 0.15406 nm and the sampling interval was 0.02°. The crystallinity is then calculated according to the formula proposed by Segal et al. (1959):

$$C_{rl}\left(\%\right)=\left[\left(I_{002}-I_{am}\right)/I_{002}\right]\times 100$$ (1)

where C_rl_ (%) is the percentage of relative crystallinity; *I*_*002*_ is the maximum intensity of (002) lattice diffraction angle (any unit); and *I*_*am*_ is the scattering intensity of amorphous background diffraction (same as I_002_ unit) when 2*θ* is close to 18°.

#### 2.3.3 Infrared Spectroscopic Analysis

The change of fiber structure was detected by Nicolet IS10 Fourier transform infrared spectrometer produced by American Nicolet company. The scanning wave number range was 4,000–500 cm^−1^. The calculation method of the relative intensity of the absorption peak is the ratio of the absorbance of the corresponding characteristic absorption peak to the absorbance of 1,372 cm^−1^; 1,372 cm^−1^ is only found in the IR of crystalline cellulose, which is a band related to crystallization (Chandel et al., 2014). The experimental results are the average of the three experimental results.

#### 2.3.4 Scanning Electron Microscope Analysis

The morphological changes of Moso bamboo samples were observed by 3400-I scanning electron microscope (SEM) produced by Hitachi, Japanese. The magnification was 500 and 1,000 times.

## 3 Results and Discussion

### 3.1 Chemical Composition Analysis After Different Pretreatment

The chemical components of Moso bamboo before and after pretreatment were determined with three samples. The results in this table were the average of the three samples.

The content of cellulose, hemicellulose, and lignin (Table 1) of Moso bamboo material were 48.59, 22.71, and 22.35%, respectively. After the LHW and MA-LHW pretreatment, the composition of cellulose, hemicellulose, and lignin in Moso bamboo has changed greatly. The percentage of cellulose in remained materials after pretreatment increased significantly, especially pretreated by MA-LHW, which has high recovery of 85.27%, making the percentage of cellulose reach 64.91%. It can be seen from the results that both LHW and MA-LHW pretreatment can effectively hydrolyze hemicellulose in Moso bamboo. After LHW pretreatment, 98.49% of hemicellulose was hydrolyzed, while 32.03% of cellulose and 34.96% of lignin were also removed. The hydrolysis rate of hemicellulose was 85.16% after MA-LHW pretreatment, but under this condition, the lignin removal rate was only 7.18%, and the cellulose recovery rate was 22.84% higher than that of LHW pretreatment.

**TABLE 1:** Chemical composition change after different pretreatments.

Samples	Solid remain	Glucan (%)		Xylan(%)		Klason lignin (%)	
	(%)	Content	Removal	Content	Removal	Content	Removal
Raw material	100.00	48.59	—	22.71	—	22.35	—
LHW	62.50	52.84	32.03	0.55	98.49	23.26	34.96
MA-LHW	63.83	64.91	14.73	5.28	85.16	32.50	7.18

### 3.2 Effects of Different Pretreatment Methods on X-Ray Diffraction

The crystallinity of lignocellulosic was once thought to be a major obstacle to its enzymatic digestibility (Puri, 2004), and some other learners think that it was a remark of the extent of material destruction (Gao et al., 2013). As show in Table 2, after being pretreated by LHW, the CrI value of Moso bamboo increased by 5.65%, and after being pretreated by MA-LHW, it increased by 14.84%. This indicates that after pretreatment by LHW and MA-LHW, most hemicellulose and part of amorphous cellulose in Moso bamboo raw materials were removed, and the proportion of cellulose with crystalline structure increased accordingly, which was consistent with previous component analysis results (Mohan et al., 2015).

**TABLE 2:** Effect of different methods on relative crystallinity of Moso bamboo.

Sample	2*θ* = 22°	2*θ* = 18.4°	Crystallization index (%)
Raw material	3,367	1,699	49.53
LHW	3,353	1,598	52.33
MA-LHW	3,147	1,357	56.88

**FIGURE 1:**
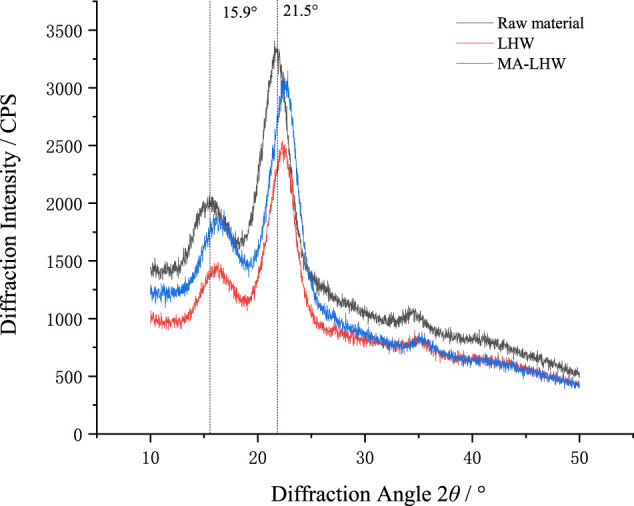
The x-ray diffraction spectra of Moso bamboo pretreated by different methods.

Generally, there are two types of natural cellulose, *I*_*α*_ and *I*_*β*_. Cellulose *I*_*α*_ and *I*_*β*_ are triclinic cells with one chain and triclinic cells with two chains, respectively (Hinterstoisser and Salmén, 1999). Figure 1 shows that the pretreatment did not change the crystal types of Moso bamboo, which all belonged to typical cellulose *I*_*α*_. At 2*θ* = 15.9°, the non-crystalline part is mainly hemicellulose and lignin peak, and at 2*θ* = 21.5°, the crystalline part is mainly *I*_*α*_ cellulose peak. However, after pretreatment with LHW and MA-LHW, the diffraction peak of Moso bamboo fiber at 21.5° shifted to 22.4°, and the peak width decreased slightly. This was also the result of the increase of the CrI value (Puri,^2004^).

### 3.3 Effects of Different Pretreatment Methods on Infrared Absorption

**FIGURE 2:**
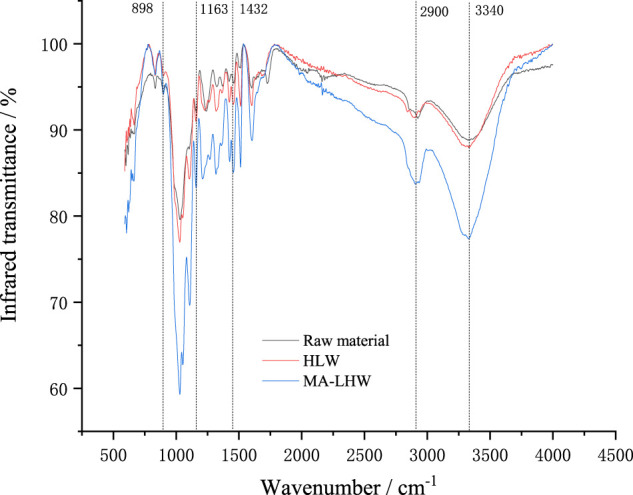
Infrared spectroscopy of Moso bamboo pretreated by different methods.

The changes in the chemical structure of materials before and after pretreatment cannot be completely identified by x-ray diffraction method alone, and other methods were needed to assist in observing the changes of other chemical groups. Moso bamboo samples being pretreated by LHW and MA-LHW were scanned by infrared spectroscopy. The results are shown in Figure 2. The structure attribution and the relative absorption intensity of the absorption peaks in infrared spectra are listed in Table 3. As can be seen from the changes of the relative absorption peak intensity, the pretreatment of MA-LHW has a more significant effect on the structure of Moso bamboo samples, which may be the result of the combined effect of physical effect and thermal effect from microwave.

**TABLE 3:** Structure attribution and relative strength of absorption peaks in IR spectra (Kim and Lee, 2005).

Wave number/cm^−1^	Response peak	Pretreatment methods		
		Raw material	Pretreated by LHW	Pretreated by MA-LHW
3,600–3,000	O-H stretching vibration	2.366	2.234	2.211
2,900	C-H stretching vibration	1.750	1.527	1.465
1,432	Cellulose CH2 bending vibration and shear vibration	0.757	1.006	1.099
1,730	Hemicellulose C=O stretching vibration	0.919	0.285	0.174
1,372	Cellulose and hemicellulose-CH bending vibration	1.000	1.000	1.000
1,163	Cellulose and hemicellulose C-O-C stretching vibration	1.577	1.578	1.429
898	β-glucoside bond vibration	1.197	0.668	0.504

The wider absorption peak near 3,340 cm^−1^ is the O-H characteristic absorption peak, which mainly comes from the glucose ring on cellulose (Sang et al., 2005). The peak near 2,900 cm^−1^ is the stretching vibration of C-H. After pretreatment with LHW and MA-LHW, these absorption peaks intensity all decreased, which indicated that the pretreatment caused a certain degree of damage to the hydrogen and methyl bonds in Moso bamboo fiber, and cellulose molecules were more likely to fracture and hydrolyze after being pretreated by MA-LHW.

Moreover, the intensities of absorption peaks at 1,163 and 898 cm^−1^ also decreased after MA-LHW pretreatment, which suggests that C-O-C bonds and β-glycosidic bonds were damaged to the same extent by MA-LHW pretreatment. C-O-C bonds and β-glycosidic bonds were the main bonds in the supramolecular chain of cellulose and glucan, and their rupture meant that the degree of polymerization of cellulose molecule decreased and this can facilitate the subsequent hydrolysis (Lino et al., 2013).

In the Moso bamboo raw material, there was an obvious absorption peak of carboxyl group at 1,730 cm^−1^, and its strength decreased significantly after pretreatment. The group was mainly the characteristic peak of hemicellulose (Schwanninger et al., 2004), so this indicated that most hemicellulose has been deacetylated, and the hemicellulose was easily damaged and degraded due to its low degree of polymerization.

However, the relative absorption peak intensity at 1,432 cm^−1^ increased after pretreatment, in which the intensity increased significantly after pretreatment of MA-LHW. This indicated that the destruction of the carbon chain structure of sample fiber generated more methylene bonds, which was a highly active reaction intermediate (Abidi et al., 2008). From Figure 2, we also noticed that C=O stretching vibration peaks of cellulose, hemicellulose, and lignin were generated at 1,050 cm^−1^ after pretreatment, which may be caused by the recondensation of some groups of cellulose, hemicellulose, and lignin destroyed under pretreatment conditions. Therefore, the pretreatment process of LHW and MA-LHW was not only a degradation process, but also caused the recombination and synthesis of some groups.

### 3.4 Effects of Different Pretreatment Methods on Surface Morphology of Moso Bamboo

**FIGURE 3:**
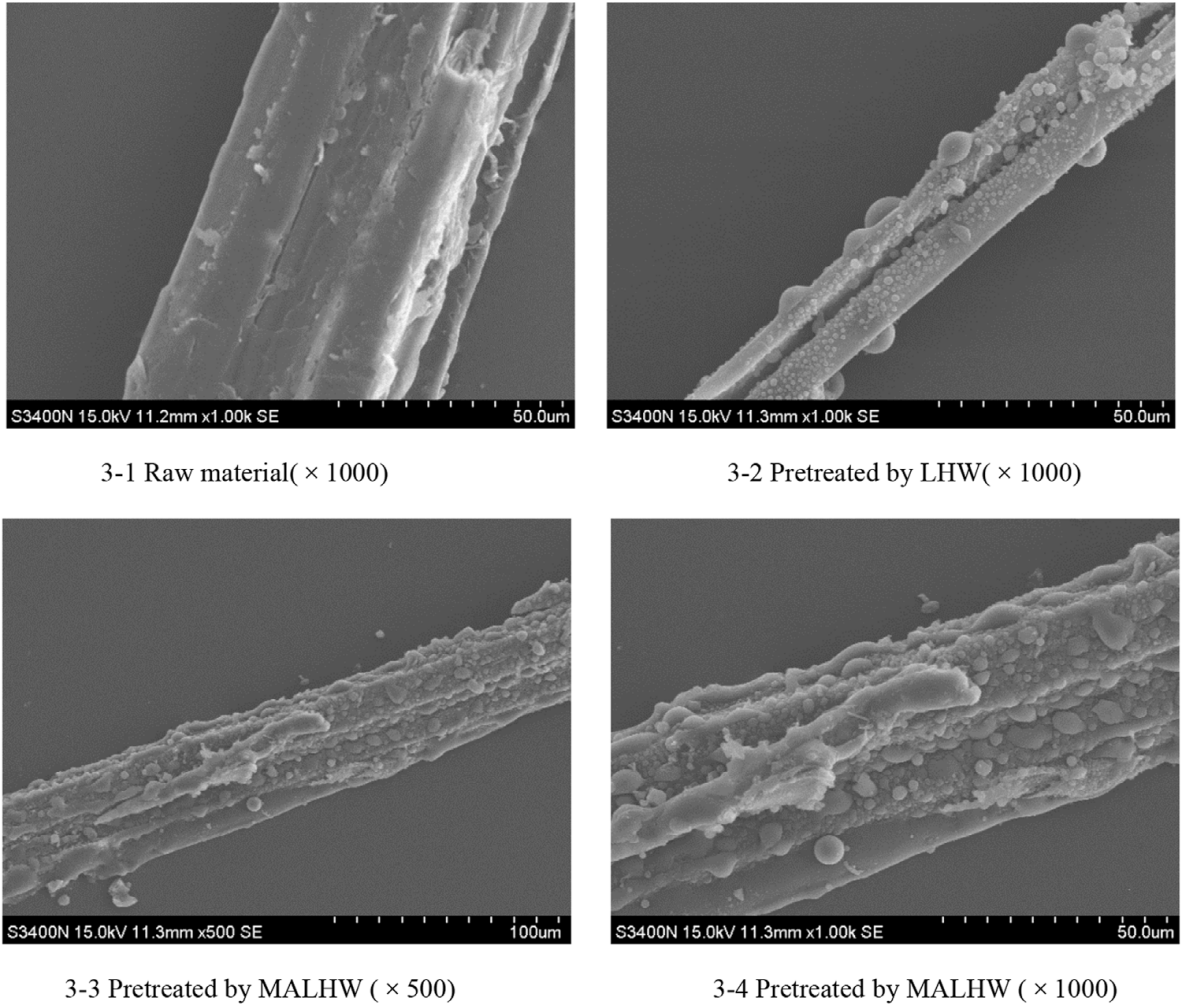
SEM picture of Moso bamboo pretreated by different methods.

It can be seen from Figure 3 of the scanning electron microscope that there are obvious differences between the surface morphology of the untreated raw materials and the treated materials, and there are also some connections. The raw material of Moso bamboo has bundle structure, which is relatively flat under the electron microscope, and there are obvious gullies, but it seems that the fibers are arranged as orderly, dense, and hard. After LHW and MA-LHW pretreatment, the structure of the whole Moso bamboo changed significantly due to the removal of hemicellulose. The smooth and orderly surface showed irregular bubbles like protrusions, which indicated that LHW and MA-LHW pretreatment had a strong erosion effect on Moso bamboo. Combined with the material composition analysis, x-ray diffraction crystallinity analysis, and IR spectrum analysis, the mass loss of Moso bamboo after MA-LHW pretreatment was more, and the changes of chemical bonds are more significant, reflecting the material morphology is more disorganized.

### 3.5 Enzyme Depolymerization of the Extracted Hemicellulose to Xylooligosaccharide

**FIGURE 4:**
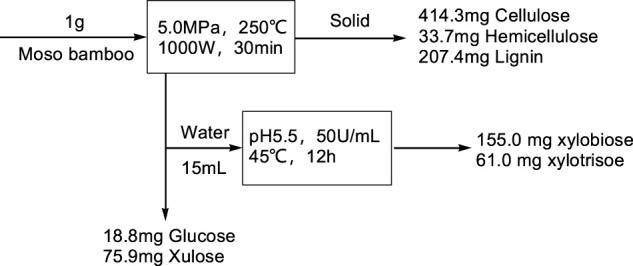
Material balance of MA-LHW pretreatment and acid hydrolysis.

The detailed material balance of MA-LHW pretreatment and enzyme depolymerization are shown in Figure 4. There was 36.17% solid loss in the whole process, and the removal rate of hemicellulose reached 85.16%. After MA-LHW pretreatment (solid-liquid ratio 5%, w/V, hydrolysis at 250°C for 30 min, power 1,000 W, pressure 5.0 MPa), 18.8 mg glucose and 75.9 mg xylose were obtained from each gram of Moso bamboo raw material. For the further depolymerization of extracted hemicellulose to obtain xylooligosaccharide, the xylanase was added into the hydrolysate with the concentration of 50 U/ml. After enzymatic hydrolysis for 20 h, the hydrolysis contained 10.33 g/L xylobiose and 4.07 g/L xylotriose. The yield of xylo-oligosaccharide reached 80.59%.

## 4 Conclusion

MA-LHW pretreatment of Moso bamboo can separate hemicellulose from lignocellulose through deacetylation reaction and enter the hydrolysate, which cannot only relieve the polymerization of hemicellulose on the structure of Moso bamboo but also facilitate the further enzymatic hydrolysis reaction. In our study, the final yield of xylooligosaccharide reached 80.59% of the theoretical yield, and the main products were xylobiose (11.83 g/L) and xylotriose (4.59 g/L). Additionally, MA-LHW pretreatment did not change the crystal types of Moso bamboo, but it caused an increase in crystallinity because most hemicellulose and part of amorphous cellulose in Moso bamboo raw materials were removed, and the proportion of cellulose with crystalline structure increased. At the same time, MA-LHW pretreatment destroyed O-H, C-H, C-O-C, and β -glucoside bonds in Moso bamboo fiber, caused by the recombination and synthesis of some groups (-CH_2_ and C=O) of cellulose, hemicellulose, and lignin destroyed under pretreatment conditions.

## Data Availability

The raw data supporting the conclusion of this article will be made available by the authors, without undue reservation.
